# An AI-empowered indoor digital contact tracing system for COVID-19 outbreaks in residential care homes

**DOI:** 10.1016/j.idm.2024.02.002

**Published:** 2024-02-10

**Authors:** Jiahui Meng, Justina Yat Wa Liu, Lin Yang, Man Sing Wong, Hilda Tsang, Boyu Yu, Jincheng Yu, Freddy Man-Hin Lam, Daihai He, Lei Yang, Yan Li, Gilman Kit-Hang Siu, Stefanos Tyrovolas, Yao Jie Xie, David Man, David H.K. Shum

**Affiliations:** a School of Nursing, The Hong Kong Polytechnic University, Hong Kong Special Administrative Region, China; b Electronic and Information Engineering, The Hong Kong Polytechnic University, Hong Kong Special Administrative Region, China; c Research Centre of Textiles for Future Fashion, The Hong Kong Polytechnic University, Hong Kong Special Administrative Region, China; d Department of Land Surveying and Geo-Informatics, The Hong Kong Polytechnic University, Hong Kong Special Administrative Region, China; e Department of Applied Mathematics, The Hong Kong Polytechnic University, Hong Kong Special Administrative Region, China; f The Jockey Club School of Public Health and Primary Care, The Chinese University of Hong Kong, Hong Kong Special Administrative Region, China; g Department of Rehabilitation Sciences, The Hong Kong Polytechnic University, Hong Kong Special Administrative Region, China; h Department of Computing, The Hong Kong Polytechnic University, Hong Kong Special Administrative Region, China; i Department of Health Technology and Informatics, The Hong Kong Polytechnic University, Hong Kong Special Administrative Region, China; j Department of Nutrition and Food Studies, George Mason University, USA; k Tung Wah College, Hong Kong Special Administrative Region, China; l Mental Health Research Centre, The Hong Kong Polytechnic University, Hong Kong Special Administrative Region, China

**Keywords:** COVID-19, Indoor contact tracing, Contact pattern, Outbreak containment, Artificial intelligence

## Abstract

An AI-empowered indoor digital contact-tracing system was developed using a centralized architecture and advanced low-energy Bluetooth technologies for indoor positioning, with careful preservation of privacy and data security. We analyzed the contact pattern data from two RCHs and investigated a COVID-19 outbreak in one study site. To evaluate the effectiveness of the system in containing outbreaks with minimal contacts under quarantine, a simulation study was conducted to compare the impact of different quarantine strategies on outbreak containment within RCHs. The significant difference in contact hours between weekdays and weekends was observed for some pairs of RCH residents and staff during the two-week data collection period. No significant difference between secondary cases and uninfected contacts was observed in a COVID-19 outbreak in terms of their demographics and contact patterns. Simulation results based on the collected contact data indicated that a threshold of accumulative contact hours one or two days prior to diagnosis of the index case could dramatically increase the efficiency of outbreak containment within RCHs by targeted isolation of the close contacts. This study demonstrated the feasibility and efficiency of employing an AI-empowered system in indoor digital contact tracing of outbreaks in RCHs in the post-pandemic era.

## 1 Introduction

Outbreaks often occurred at residential care homes (RCHs), restaurants, schools, and residential buildings during the COVID-19 pandemic (Adam et al., 2020; Huang et al., 2021; Liu et al., 2021; Stockdale et al., 2022). This highlights the need to enhance infection control measures inside crowded indoor environments. Social distancing could be one of the effective measures, but it is difficult to sustain, particularly in the post-pandemic era. Although most COVID-19 vaccines remain effective in reducing the risks of severe infections and mortality, their impact on the transmission of SARS-CoV-2 variants is somewhat limited (Andrews et al., 2022; Stokel-Walker, 2022). The COVID-19 pandemics has emphasized the need to design an efficient and sustainable contact tracing system in high-risk locations as part of future pandemic preparedness plans.

Digital contact tracing at the population level using GPS-based mobile applications has been applied in different countries or regions, including Hong Kong (Braithwaite et al., 2020). These mobile applications adopted two types of architectures, namely, centralized and decentralized architectures (Shubina et al., 2020). The centralized apps collect location data and other personally identifiable information in a central server, where the government agencies store and manage the user's data. The decentralized apps allow the users to keep the contact data locally on their mobile phones and use privacy-preserving protocols to prevent them from sharing any personally identifiable information with the central server unless the user self-reported being infected. Some apps chose to track person-to-site proximities (such as the LeaveHomeSafe app in Hong Kong), and others collected person-to-person contact data. Nevertheless, there are limited data on temporal and spatial connectivity of people inside indoor environments. Real-world data on the duration, proximity, and temporal patterns of both person-to-person and person-to-site contacts of COVID-19 cases, however, would allow us to better define close contact and provide essential insights on improving contact tracing and efficient quarantine to prevent virus transmission. A better understanding of the indoor transmission patterns of SARS-CoV-2 would also allow policymakers to promote evidence-based contact tracing strategies, with the aim to effectively containing the indoor transmission of COVID-19, particularly in high-risk areas such as RCHs.

This study was aimed to develop an AI-empowered contact-tracing system for automatically collecting high-resolution contact data of residents and staff in RCHs with careful preservation of personal privacy. Previous studies have used mobile phones with Wi-Fi, 4G/5G and GPS signals for indoor positioning (Kim et al., 2021), which may not be applicable to RCHs due to privacy concerns and large energy consumptions. Here we adopted a low-energy Bluetooth technology to achieve accurate indoor localization and tracking of movements of resident, staff and daycare users. In this paper, we reported the structure and algorithms adopted in this system. We also conducted an in-depth analysis on the contact patterns collected from two RCHs in Hong Kong and the outbreak data in one of them, to demonstrate the implications of this system on COVID-19 outbreak containment.

## 2 Results

### 2.1 Participant characteristics

All the staff, day care users and residents in two local RCHs in Hong Kong were invited to participate in the study. In Hong Kong, most RCHs were operated by non-governmental organizations (NGO) and private entities to provide residential care services for the elderly and the people with disabilities. In RCH-A (an elderly home), 58 out of the 78 individuals signed the informed consent form, with a response rate of 74.4%. However, one staff and two elderly participants withdrew from the study, and one participant passed away, so the final sample size was 54 participants. Of these, 28 (51.8%) were staff, 17 (31.5%) were day care users, and 9 (16.7%) were residents. Overall, 22% of participants were men (7.1% in staff, 38.5% in day care users and residents). The age range of the participants was between 25 and 94 years, with a mean (*SD*) of 51.2 years (12.5) for staff and 84.7 years (7.5) for elderly participants. In RCH-B (a RCH for both elderly and disabilities, without day care units), after excluding a dropout case, 102 participants joined and completed the study, with a response rate of 52.9%. The sample consisted of 32 (31.4%) staff and 70 (68.6%) residents, 59.8% of whom were female. The age range was between 30 and 102 years, with a mean (*SD*) of 46.3 years (9.9) for staff and 78.5 years (17.1) for residents.

### 2.2 Contact patterns

Fig. 2 shows the daily contact hours (6am–10pm) between staff members and residents in two RCHs. In RCH-A, the average duration of contact between residents was longer than the contact duration between staff members. In weekdays residents tended to have more activities which resulted in longer contact hours than in weekends, while the day care users showed an opposite pattern. The weekday-weekend difference was statistically significant for resident-resident, day care user-day care user, staff-resident, and day care user-resident pairs (p < 0.05), but not significant for staff-staff and day care user-staff pairs. In RCH-B, no differences of these contact hours were found statistically significant between weekends and weekdays, for staff-staff, resident-resident and staff-resident pairs (p > 0.05).

**Fig. 1:**
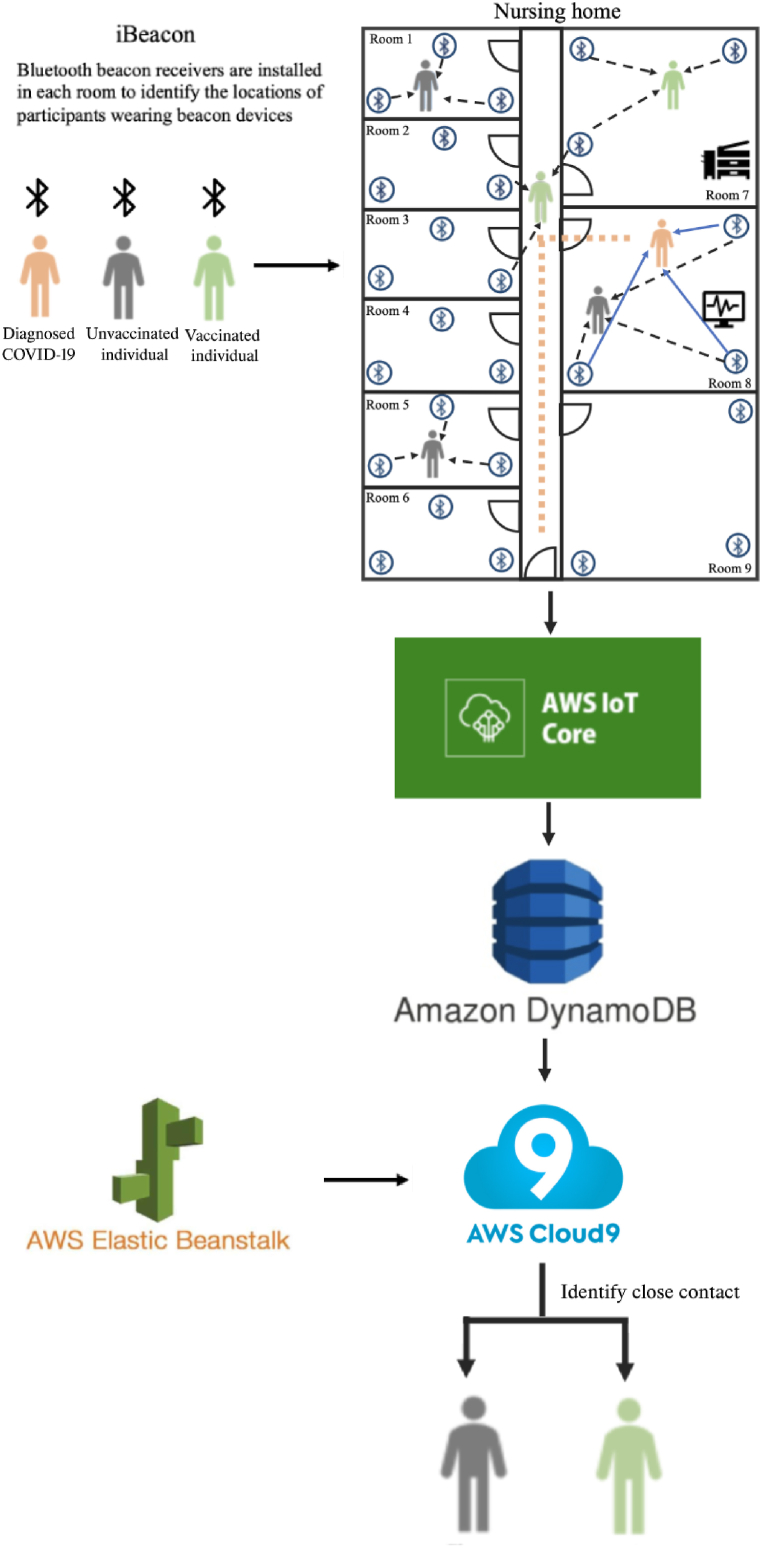
Architecture of the system.

**Fig. 2:**
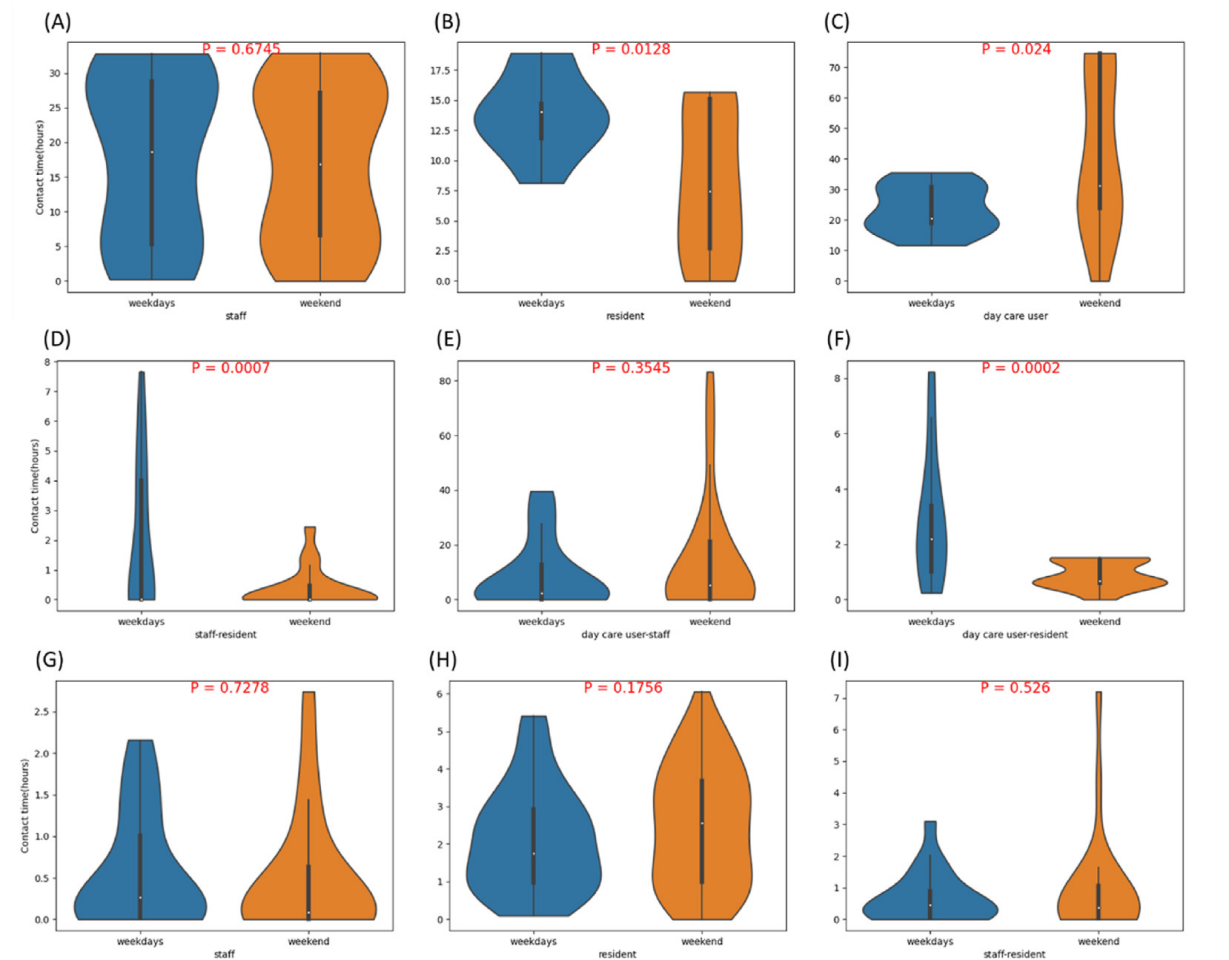
Plots of daily contact hours (A) between staff, (B) between residents, (C) between day care users, and (D) between staff and residents, (E) between staff and day care users, (F) between residents and day care users in RCH-A; (G) between staff, (H) between residents, and (I) between staff and residents in RCH-B, in weekdays (Monday to Friday) and weekends (Saturday to Sunday), respectively. Daily data were collected during 6am to 10pm every day. Each data point represents the sum of daily contact hours with all the other residents/staff/day care users by one participant. *P*-values of the two-sided t-tests between weekday and weekend contact hours are shown in each figure.

### 2.3 Investigation of a COVID-19 outbreak

During the study period, there was a COVID-19 outbreak in one of enrolled RCHs (RCH-B). As required by the infection control regulations placed by the government at that time, all staff and residents on the same floor were moved to the quarantine centers two days after diagnosis of the index case (R14 on Day 0), and five more cases (R2 and R9 on Day 2; R12 on Day 4; R1 and R10 on Day 5) were confirmed by rapid antigen tests (RAT) within six days. Of 14 residents on this floor, six participants were tested positive for SARS-CoV-2 by RAT during the entire outbreak. No significant difference was found between SARS-CoV-2 tested positive and negative participants on this floor (Table 1). All staff and residents in RCH-B who took the compulsory RAT tests for SARS-CoV-2 on the same day when the index case was diagnosed, but no cases were found on the other floors.

**Table 1:** Comparison of the participants in RCH-B who were tested negative and positive for SARS-CoV-2 during the study period.

	SARS-CoV-2 positive (n = 6)	SARS-CoV-2 negative (n = 8)	p-value a
Age (years)	54.0 ± 7.0	46.4 ± 10.5	*0.244*
Gender			*0.277*
Female	2 (33.3%)	2 (25.0%)	
Male	4 (66.7%)	6 (75.0%)	
Smoking history			*0.429*
Ever smoker	1 (16.7%)	0	
Non-smoker	5 (83.3%)	8 (100%)	
Received influenza vaccine last year	5 (83.3%)	8 (100%)	*0.429*
COVID-19 vaccination			*0.369*
Primary and booster	6 (100%)	7 (87.5%)	
Primary two doses	0	1 (12.5%)	
Type of COVID-19 vaccination			*1.000*
Sinovac	6 (100%)	7 (87.5%)	
BioNTech	0	1 (12.5%)	
Health records			
Hypertension	3 (50.0%)	1 (12.5%)	*0.245*
Liver diseases	1 (16.7%)	0	*0.429*
Diabetes	1 (16.7%)	1 (12.5%)	*1.000*
Have regular doctor visits	6 (100%)	6 (75.0%)	*0.473*
Self-rated health			*0.277*
Very well	0	0	
Very good	2 (33.3%)	6 (75.0%)	
Good	4 (66.7%)	2 (25.0%)	
Normal	0	0	
Bad	0	0	
Have visitors	3 (50.0%)	3 (37.5%)	*0.640*

a Kruskal-Wallis test was conducted for continuous variables, and Fisher exact test for categorical variables.

**Fig. 3:**
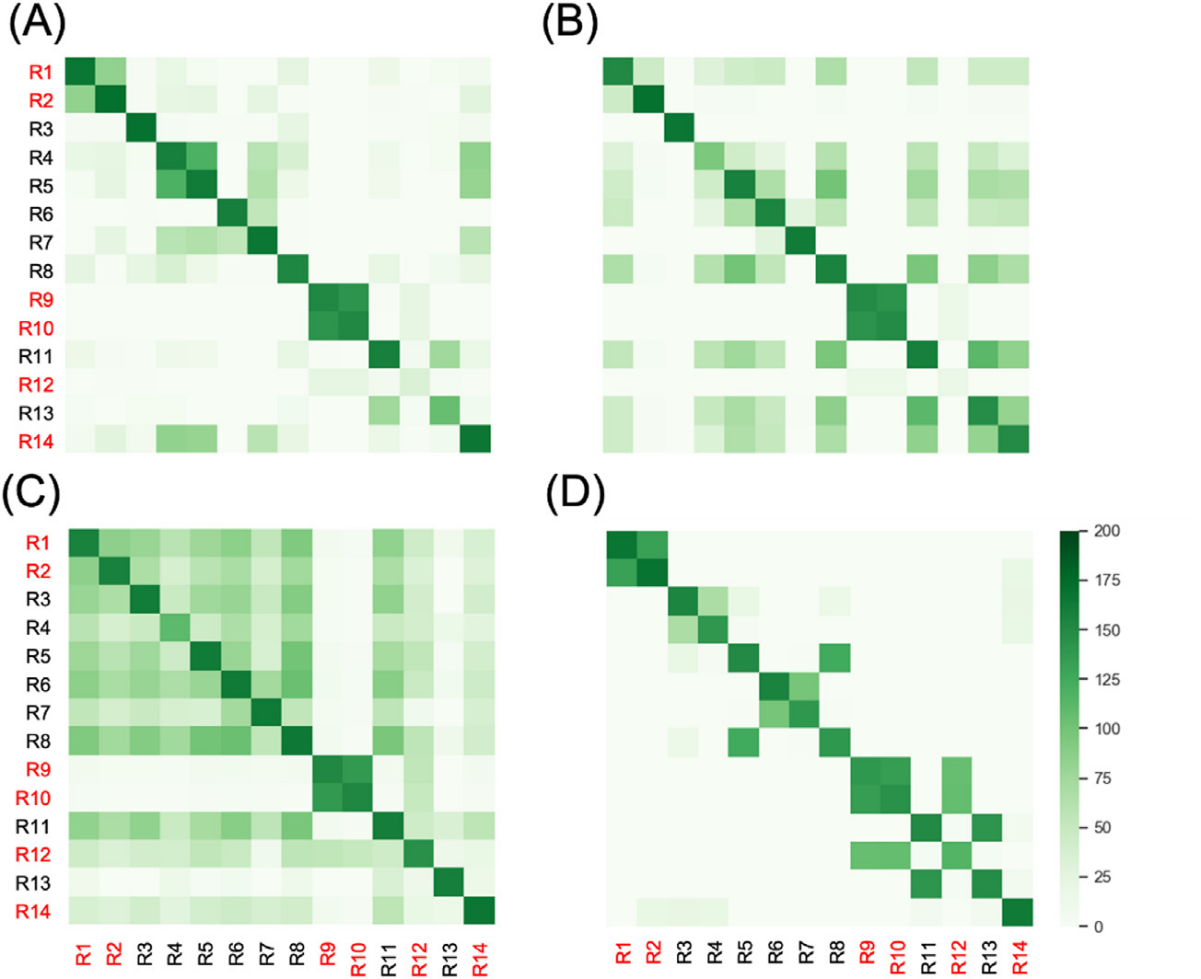
Heat map of daily contact duration (in minutes) between participants, on (A) the day of index case diagnosis, (B) one day, (C) two days, and (D) three days prior to that day. R14 is the index case. R1, R2, R9, R10 and R12 are secondary cases (highlight in red). The diagonal line shows the total duration of staying in the residential care home in the exposure period.

We compared the direct contact duration on the day of index case diagnosis and up to three days prior (Fig. 3). R1 and R2 were staying in the same rooms with longer contact duration, similarly for R9 and R10. There is no significant difference in contact durations with the index case observed between positive cases and negative cases.

### 2.4 Simulation of different outbreak containment strategies

**Fig. 4:**
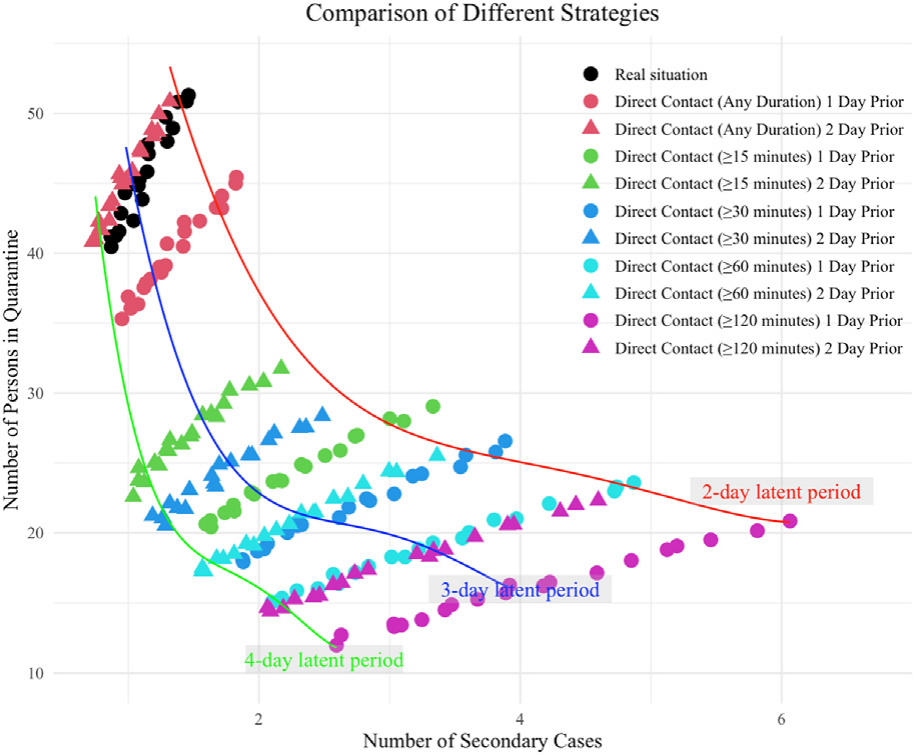
Simulation of total secondary cases under different outbreak containment strategies, including quarantine of close contacts who had direct contact with the index case at the threshold of accumulative 15, 30, 60 and 120 min or no threshold, up to two days prior to the diagnosis date of the index case. A multinomial regression line is fitted to the data points under different assumptions of latent period. The contact duration data were from the data collected during the outbreak in RCH-B.

We simulated the different outbreak containment strategies including quarantine of close contacts who had direct contact with the index case at the threshold of accumulative 15, 30, 60 and 120 min or no threshold, up to two days prior to the diagnosis date of the index case. These scenarios were compared with the real situation that all residents and staff on the same floor were quarantined for 7 days. The final size of outbreak decreased as more people were quarantined, with an L shape turning at the threshold of ≥15min one day prior and ≥30min one day prior to the diagnosis date of the index case (Fig. 4). The graph demonstrates that as the incubation period extends, the curve's slope, which represents the number of isolated individuals versus the number of infected individuals, increases. This implies that to achieve fewer infections, more individuals need to be isolated.

## 3 Discussion

In this study, we developed and tested an AI-empowered indoor digital contact-tracing system for COVID-19 outbreaks and tested it in two RCHs. Using lower-energy Bluetooth technologies, we were able to monitor people's indoor movements in real-time while ensuring privacy and data security. The system demonstrated its potential in rapidly identifying close contacts and high-risk areas, aiming to effectively contain outbreaks in RCHs, especially in the current context where almost all control measures have been lifted. This system has the potential to greatly benefit institutionalized populations with minimal manpower and maintenance costs. Given the high transmissibility and immune breakthrough ability of the emerging SARS-CoV-2 variants (Zheng et al., 2022), our AI-empowered contact tracing system could serve as an effective and convenient infection control tool, with minimal interference to daily life of RCH residents. Compared with previous studies, our system only uses low-energy Bluetooth technologies, which further reduces the maintenance cost and compliance of users. In addition, this system can be easily expanded to other settings with high risks of respiratory disease outbreaks, such as schools and restaurants.

Another advantage of our system is preservation of personal data and privacy. A few existing automatic digital contact tracing systems adopt proximity-based technologies, such as Bluetooth, in which signals are directly detected by nearby smart devices (e.g. smartphones) instead of the exact geographic location (Grekousis & Liu, 2021). However, such systems have certain restrictions. Firstly, the users may not want to turn on the Bluetooth of their smart devices all the time due to battery drainage. Secondly, people may not carry their smart devices along when having their normal indoor activities. Thirdly, such systems need an additional application to be installed on the smart devices. Users may be hesitant to install the application on their smart devices because of privacy issues. All of these affect the suitability to construct a sustainable and desirable contact tracing system. Our indoor tracking system overcomes these limitations. The beacons battery consumption are low, with an estimated battery life of three years. In addition, the portable beacon devices (either card-type or wrist-type) are convenient for everyday use. They are light and waterproof, which would not disturb the daily life. Data collection using our device is completely automatic and passive. The gateways capture only Bluetooth advertisement packets emitted by any nearby beacons. These packets do not reveal any personally identifiable information about the user and hence no privacy issues aroused. Furthermore, the beacon devices remain completely anonymous to the data analysts at the analysis phase. It's important to note that our system, designed for identifying cases and controlling outbreaks, does not account for personal behaviors and is represented on an aggregated scale. During an outbreak, case investigations into exposure history and individual risk factors (such as age, comorbidities, etc.) are still necessary to complement this AI system.

Hong Kong had adopted the zero-COVID policy at the early phase of the pandemic and remained as the place least affected by the COVID-19 until the end of 2021. However, Hong Kong had the highest case-fatality rate in the world during the Omicron wave in early 2022, due to a low vaccination rate in the elderly and low population immunity (Chen et al., 2022). Nearly 50% of COVID-19 related deaths during this wave occurred in RCHs. Although the vaccine uptake rates in the 60+ age group have improved to 81.2% for one dose and 76.9% for booster (as of June 12, 2023), the emergence of new XBB variants continue to remind us that vaccination alone cannot offer sufficient protections to this vulnerable population. Therefore, our AI-empowered system could have a great potential of implementation in the post-pandemic era with minimal requirement of work force and careful preservation of personal privacy. Designated persons from nursing homes who distribute the beacons have no access to the contact tracing data. They are allowed to access the contact data only if one participant is diagnosed with COVID-19 or other infectious diseases of concern. The persons who manage the data on server do not know the beacon users. So the privacy of the participants is well protected while the efficiency in data collection and processing is also maintained.

Our study has some limitations. First, the received RSSI signals are affected by many other factors, such as the refraction of metal and interference from other Bluetooth devices. Second, we only collected the data from two RCHs with a small sample size, and further studies are still needed to explicit the clear definition of close contacts. Third, if someone forgot to wear the devices, the tracking information would be lost. This could be solved by tracking devices with abnormal static signals and emit alerts from the master system to the staff of the RCH. Fourth, Hong Kong has highly condensed living condition and most RCHs are crowded without any outdoor open space. Therefore, the findings of contact pattern data may not be generalized to other countries or regions. Nevertheless, the findings of this study demonstrate that the AI-empowered system can achieve satisfactory efficiency and accuracy in indoor digital contact tracing of outbreaks within RCHs in the post-pandemic era.

The development of an AI-empowered system for contact tracing in residential care homes presents a promising approach for effective outbreak containment. Field studies demonstrated the feasibility and efficiency of this system, and simulation studies showed the importance of targeted quarantine of close contacts. At the same time, this system can be used in other disease outbreaks, simply by adding tests for these viruses in active surveillance.

## 4 Methods

### 4.1 The system architecture

Fig. 1 shows the architecture of this digital contact-tracing system. The system has three key components: 1) indoor positioning data collection via wearable low-energy Bluetooth beacons and gateways (i.e. signal receivers), 2) AI algorithms stored at the cloud server for automatic cleaning and processing of indoor positioning data, and 3) a web portal for data enquiries and output visualization. Wearable Bluetooth beacons have better performance in accuracy and efficiency, and less energy consumptions for indoor walking path tracking, than the GPS-based mobile applications. As personal information is not associated with these Bluetooth beacons, concerns regarding privacy and data security are minimum (Li et al., 2018; Welch, 2021, pp. 1–3).

In this system, each room has a minimum of three gateways (AB BLE Gateway 4.0, Beijing April Brother Technology Co., Ltd) installed, enabling precise positioning of individuals within rooms through trilateration. The wearable beacon devices (ABN02, Beijing April Brother Technology Co., Ltd) transmit their received signal strength indication (RSSI) to these gateways at a frequency of 3 s (Wang et al., 2011). Subsequently, the Amazon Web Services (AWS) cloud server automatically collected and stored these RSSI signals via the Message Queuing Telemetry Transport (MQTT) protocol.

All RSSI signals received from gateways were automatically stored in the AWS server and first processed by localization algorithms. Detailed information about equipment calibration, data validation and noise reduction can be found in Supplementary Note. The AWS server computed and archived the beacon coordinates and timestamps based on these localization algorithms and the known locations of the gateways. We developed a dedicated web portal to facilitate data visualization and interpretation, by providing a map of beacon movement paths, a list of high-risk zones and close contacts of a COVID-19 case (if any). In the event of a COVID-19 outbreak within a RCH, the portal provides crucial information to enable the RCH manager to initiate appropriate responses immediately.

As RSSI signals from one beacon were received by multiple gateways at the same time, we first identified the real-time location of each beacon as the room with the highest number of records. After acquiring the timestamps in seconds, we combined all timestamps into time intervals for each beacon. To reduce data noise, if two adjacent timestamps of one beacon exceeded 30 s, we did not combine them into one time interval. Subsequently, we searched for overlapping beacon time intervals, ultimately determining the contact duration of people who were carrying those beacons and signals were picked up by same gateways.

### 4.2 Field studies

Since the beginning of the pandemic, the Hong Kong government had adopted strict public health measures and maintained low level of COVID-19 activities before the emergence of the omicron variants in early 2022 (Hong Kong Special Administrative Region Government, 2022; Mahtani & Yu, 2022). As of 25 Jan 2023, there were 2 863 475 COVID-19 cases in the latest epidemic situation of the 5th wave in Hong Kong and 13 120 deaths, among whom 86.8% were the elderly aged 70+ years and 47% occurred in residual care homes (Kong Special Administrative Region Government, 2022). In response, until 28 Feb 2023 all residents and staff in RCHs were required to undergo regular RT-PCR tests for SARS-CoV-2, and RCH residents were sent to quarantine centers if there was an outbreak inside RCHs, which had greatly interfered their daily life and caused additional stress in both residents and staff.

This study was approved by the PolyU Institutional Review Board (PolyU IRB) (Reference Number: HSEARS20210924002). We recruited two RCHs to test the feasibility of this digital contact-tracing system. After obtaining the written consent from residents (or their guardians) and staff, we installed the gateways on wall or ceiling in the RCHs at the height of one to 3 m. One designated person at each RCH distributed Beacons to the participants and kept the record, in order to avoid revealing their identities to the researchers. After two weeks of data collection, the participants filled in a questionnaire, to collect their demographics, chronic conditions, self-report health status, COVID-19 vaccination history and previous infections. For residents, there were extra items about whether they met any visitors during the study period. The staff participants were asked to report the dates on duty and taking sick leave (including diagnosed COVID-19 during the data collection period).

### 4.3 Simulation models

We used an agent-based model (ABM) to simulate the spreading of COVID-19 infections in a nursing home setting over a period of seven days, under the assumptions of different outbreak containment strategies. ABM has been widely adopted in infectious disease modelling due to its capability of capturing complex system dynamics and heterogeneity among individuals (Meyers, 2007). This simulation utilized the contact pattern data of 73 participants from the RCH B, where a COVID-19 outbreak occurred during the data collection period.

We examined three different scenarios. The first scenario represented the real-life (status quo) situation, in which all individuals living on the same floor as the first confirmed case were isolated. In the second scenario, only those who had direct contacts with the first confirmed case on the day before onset were isolated, with the threshold of cumulative contact time set at 0, 15, 30, 60, and 120 min. The third scenario assumed that those who had direct contacts with the first confirmed case up to two days before onset, again with thresholds set at 0, 15, 30, 60, and 120 min. Based on the literature (Wu et al., 2022), the latent period for the disease was set to range from one to three days and the daily probability of transition into the infectious period was the reciprocal of the incubation period (in days). The infectious period was set to the maximum of three days and the daily infection rate was calculated using the following formula:

$$P=\left(\frac{1}{1+e^{-\beta_{1}(t-t_{fixed})-\beta_{2}\alpha }}\right)\cdot \left(1-(1-\beta_{3})\cdot \theta_{1}\right)\cdot \left(1-(1-\beta_{4})\cdot \theta_{2}\right)$$

Where t denotes the total duration of contact, quantified in seconds, which reflects the time spent by an individual in the same room with all infected persons over the course of a day, α denotes the age of the contacted, $\theta_{1}$ denotes the past infection history, and $\theta_{2}$ denotes the vaccination status. $\beta_{1}$, $\beta_{2,}{\;\beta }_{3}\text{,}$ and $\beta_{4}$ denotes the corresponding effect coefficients. Detailed information about parameter assumptions and references is shown in Supplementary Table 1.

## CRediT authorship contribution statement

**Jiahui Meng:** Data curation, Formal analysis, Writing – original draft, Writing – review & editing. **Justina Yat Wa Liu:** Conceptualization, Writing – review & editing. **Lin Yang:** Conceptualization, Formal analysis, Funding acquisition, Investigation, Methodology, Supervision, Writing – original draft, Writing – review & editing. **Man Sing Wong:** Methodology, Writing – review & editing. **Hilda Tsang:** Data curation, Project administration, Writing – review & editing. **Boyu Yu:** Formal analysis, Validation, Writing – review & editing. **Jincheng Yu:** Formal analysis, Writing – review & editing. **Freddy Man-Hin Lam:** Writing – review & editing. **Daihai He:** Formal analysis, Writing – review & editing. **Lei Yang:** Writing – review & editing. **Yan Li:** Writing – review & editing. **Gilman Kit-Hang Siu:** Writing – review & editing. **Stefanos Tyrovolas:** Conceptualization, Writing – review & editing. **Yao Jie Xie:** Writing – review & editing. **David Man:** Funding acquisition, Writing – review & editing. **David H.K. Shum:** Funding acquisition, Writing – review & editing.

## Declaration of competing interest

The authors declare that they have no known competing financial interests or personal relationships that could have appeared to influence the work reported in this paper.
