# Supplementary material for: An AI-empowered indoor digital contact tracing system for COVID-19 outbreaks in residential care homes
Source: Infect Dis Model. 2024 Feb 10;9(2):474–82. doi: 10.1016/j.idm.2024.02.002 (PMC10885586; doi:10.1016/j.idm.2024.02.002)
Supplement: Multimedia component 1 [file mmc1.docx]

# **Supplementary Note**

#

# **Localization algorithm**

We adopted the weighted center localization (WCL) algorithms to calculate the location of beacons based on the RSSI data. We selected a threshold of RSSI > -90dbm to filter out noise^1^, as our test data indicated that this range corresponds to a distance of >10 meters. A Kalman filter was applied to reduce the error range of location data to approximately two meters^2^. WCL algorithm was based on the highest three RSSI values of the same beacon collected by different gateways in the five-second sliding time window^3^. We calculated the buffer zone of each beacon based on each RSSI value, and estimated the real-time location of beacons by overlapping regions of these buffer zones. In order to assess the validity of the proposed algorithms, ground truth data were obtained using light detection and ranging (LiDAR) technology^4^, which has been demonstrated to provide the accurate location data but not suitable for real-time tracking.

# **Noise reduction**

Since some noises in the RSSI values and natural causes cause the noise, the noise is in Gaussian distribution, so Kalman Filter is a suitable method to eliminate them. We use two Kalman filters for the system. The first one is the 1D-Kalman Filter, which is in the transmission between the RSSI value and the distance to eliminate raw noises. The second one is the 2D-Kalman Filter to smooth the moving path after getting the coordinate.

According to Welch G. and Bishop G.,^5^ there are two steps in the Kalman Filter, time update (Predict) and measurement update (Correct). Through the Kalman Filter, we can get a combined estimated value and measurement value.

In the process of reducing noise, we use the Kalman Filter twice. The result is in Supplementary Fig. 1 and Supplementary Fig. 2. In the graph of the 1D-Kalman Filter (Supplementary Fig. 1), the blue line is the range of raw data, and the orange line is the filtered data. Through 1D-Kalman Filter, we successfully restrict the RSSI value from -45 ~ -85 to -56 ~ -62, which is easier to get a more precise distance. In Supplementary Fig. S2, red points are the location of seven gateways, and blue points are the calculating coordinate result. In the test, the movement of Beacon is in the room. However, we can find that some blue points are outside the room, but after 2D-Kalman Filter, the movement path matches our tests.

# **Equipment calibration**

To test the accuracy of our algorithms, we use the light detection and ranging (LiDAR) scan images as ground truths. Supplementary Fig. 3 and Fig. 4 show a representative sample of the testing procedure conducted in an indoor setting. By comparing the location data derived from LiDAR measurements with those acquired from the artificial intelligence (AI) system, the AI algorithms were subsequently calibrated and validated. The estimated error range for location data was determined to be approximately 2 meters.

**Supplementary Figure 1. Sample of 1D-Kalman Filter.**


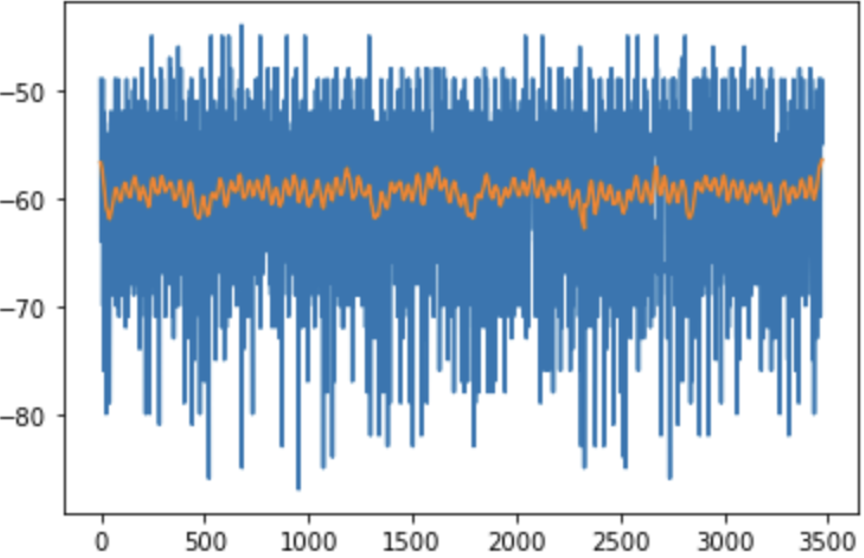


**Supplementary Figure 2. Sample of 2D-Kalman Filter.**


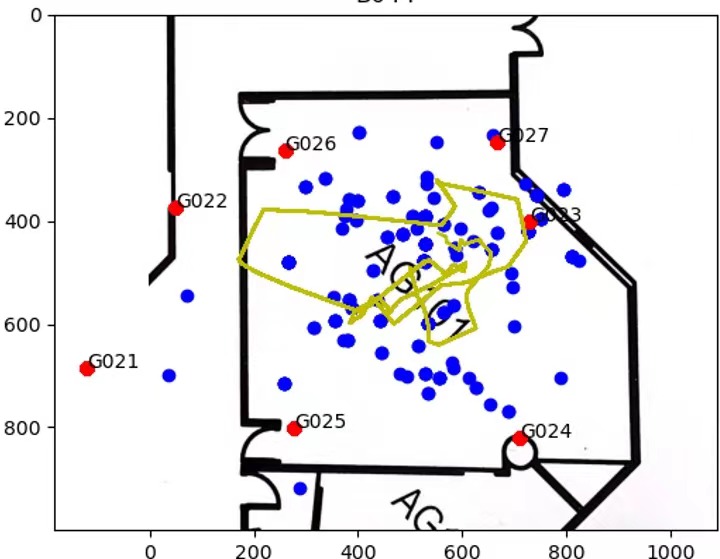


**Supplementary Figure 3. A sample floor plan output of the LiDAR scanner in an indoor setting.**

**
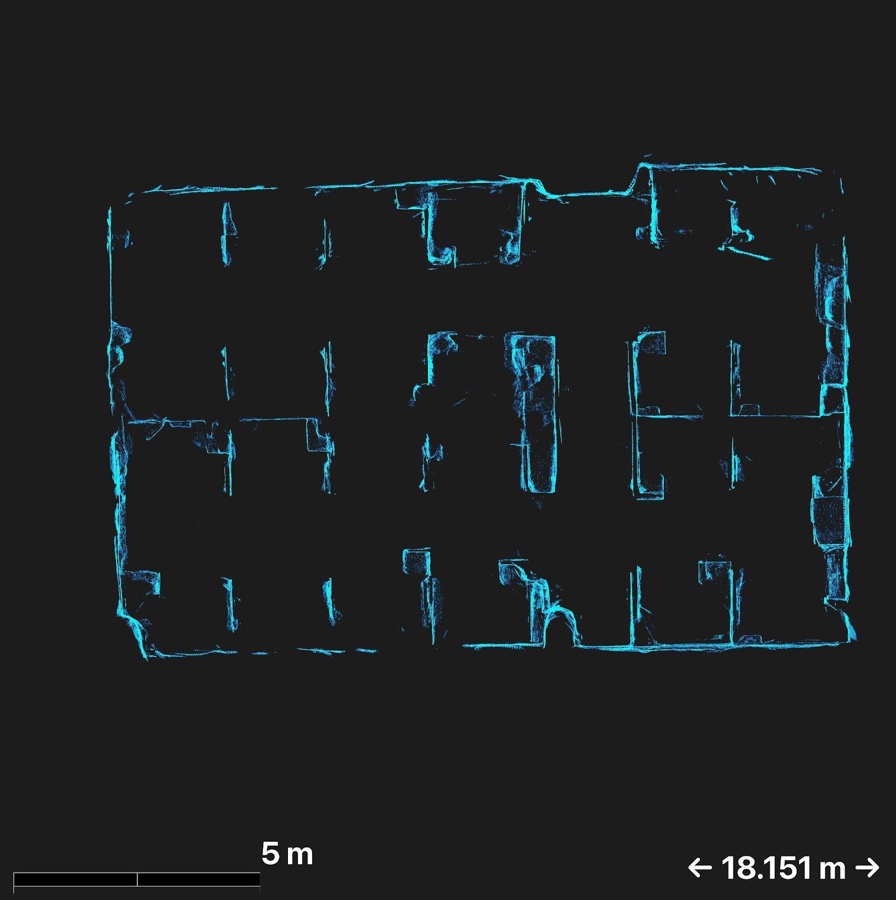
**

**Supplementary Figure 4. Walking path visualized by the LiDAR,** **with movement track of a Beacon card shown in green colour.**


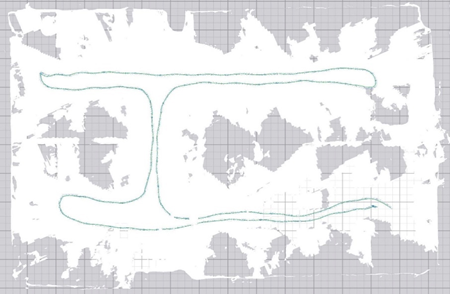


**Supplementary Table 1. Summary of parameter assumptions in the agent-based model.**

| **Parameters** | **Value** | **Explanations** | **References** |
| --- | --- | --- | --- |
| Latent/incubation period | 2-4 days | We assume equal latent and incubation periods. The usual incubation period is 3 days. However, considering the close proximity in nursing homes, there may be environmental factors that can go undetected. Therefore, we have appropriately shortened the incubation period, starting from one day and gradually increasing it to three days as a simulated condition. | Tanaka, H,, Ogata, T,, Shibata, T., et al., Shorter Incubation Period among COVID-19 Cases with the BA.1 Omicron Variant. *International Journal of Environmental Research and Public Health* **19**, 10 (2022). |
| Infectious period | 3 days | Given the fact that all RCH staff and residents in nursing homes underwent daily tests for SARS-CoV-2, we assumed a relatively shorter infectious period. |  |
| $\beta_{1}$ | 0.002  ${seconds}^{-1}$ | By fitting the actual data of infections in the nursing home and the contact time between individuals. | Infectious period |
| $\beta_{2}$ | 0.005  ${year}^{-1}$ | By fitting the actual data of infections in the nursing home and the contact time between individuals. |  |
| $\beta_{3}$ | 0.9 | The efficacy of the vaccine against the Omicron variant appears to be significantly diminished. However, our simulation models suggest some degree of residual protection, with individuals who have received the complete vaccination course demonstrating approximately 10% resistance to this variant. | McMenamin, M.E., Nealon, J., Lin, Y., et al., Vaccine effectiveness of one, two, and three doses of BNT162b2 and CoronaVac against COVID-19 in Hong Kong: a population-based observational study. *Lancet Infectious Diseases* **22**, 1435–1443 (2022). |
| $\beta_{4}$ | 0.9 | The infection probability of individuals with previous COVID-19 infections is same as that of individuals who were fully vaccinated. |  |
| $t_{fixed}$ | 3600 seconds | By fitting the actual data of infections in the nursing home and the contact time between individuals. |  |

**References in appendicies**

1. Li GQ, Geng EX, Ye ZY, Xu YJ, Lin JZ, Pang Y. Indoor Positioning Algorithm Based on the Improved RSSI Distance Model. *Sensors-Basel* **18**, 9 (2018).

2. Welch G. Kalman Filter. Computer Vision, A Reference Guide: 1–3 (2021).

3. Wang J, Urriza P, Han Y, Cabric D. Weighted Centroid Localization Algorithm: Theoretical Analysis and Distributed Implementation. *IEEE Transactions on Wireless Communications* **10**, 3403–3413 (2011).

4. Luetzenburg G, Kroon A, Bjork AA. Evaluation of the Apple iPhone 12 Pro LiDAR for an Application in Geosciences. *Sci Rep-Uk* **11**,1 (2021).

5. Welch G, Bishop G. An introduction to the Kalman filter. 1995: 127–32.
